# Combination of Genetics and Nanotechnology for Down Syndrome Modification: A Potential Hypothesis and Review of the Literature

**Published:** 2019-03

**Authors:** Alireza TAFAZOLI, Farkhondeh BEHJATI, Dariush D. FARHUD, Mohammad Reza ABBASZADEGAN

**Affiliations:** 1. Department of Analysis and Bioanalysis of Medicine, Faculty of Pharmacy with the Division of Laboratory Medicine, Medical University of Bialystok, Bialystok, Poland; 2. Department of Endocrinology, Diabetology and Internal Medicine, Clinical Research Center, Medical University of Bialystok, Bialystok, Poland; 3. Genetics Research Center, University of Social Welfare and Rehabilitation Sciences, Tehran, Iran; 4. School of Public Health, Tehran University of Medical Sciences, Tehran, Iran; 5. Department of Basic Sciences, Iranian Academy of Medical Sciences, Tehran, Iran; 6. Medical Genetics Research Center, Mashhad University of Medical Sciences, Mashhad, Iran

**Keywords:** Down syndrome, CRISPR/Cas9, Designed DNA construct, Poly D L-lactide-co-glycolide (PLGA), Nano-carrier, Chromosome 21 inactivation

## Abstract

Down syndrome (DS) is one of the most prevalent genetic disorders in humans. The use of new approaches in genetic engineering and nanotechnology methods in combination with natural cellular phenomenon can modify the disease in affected people. We consider two CRISPR/Cas9 systems to cut a specific region from short arm of the chromosome 21 (Chr21) and replace it with a novel designed DNA construct, containing the essential genes in chromatin remodeling for inactivating of an extra Chr21. This requires mimicking of the natural cellular pattern for inactivation of the extra X chromosome in females. By means of controlled dosage of an appropriate Nano-carrier (a surface engineered Poly D, L-lactide-co-glycolide (PLGA) for integrating the relevant construct in Trisomy21 brain cell culture media and then in DS mouse model, we would be able to evaluate the modification and the reduction of the active extra Chr21 and in turn reduce substantial adverse effects of the disease, like intellectual disabilities. The hypothesis and study seek new insights in Down syndrome modification.

## Introduction

Down syndrome (DS) is one of the most famous and prevalent genetic diseases in medical history (1, 2). The frequency of DS is one in every 660 individuals in different populations (3). Although there are some verified prenatal tests, which indicate the presence of the disease with great accuracy and specificity (4–6), it is still one of the most worldwide consideration to control by health care providers and the affected people themselves, especially in the Middle East and third world countries. Moreover many previous studies have tried to introduce prevention/treatment issues. To date, many treatments or modifying procedures including general and symptomic interventions have been suggested by investigators (7–11). Here we proposed a method, which can potentially reduce the main effects and clinical features in DS significantly.

Referring to published papers, we can find numerous studies about more important genes that show higher impact in creating DS clinical manifestations. Investigations on Down patients with partial trisomy and/or mosaicism form, revealed a 1.6 Mb critical region on chromosome 21q22 with highest amount of transcription in DS (12, 13). This area is called DS Critical Region (DSCR). Most of the genes, located in DSCR are related to development of the brain and play an essential role in learning and cognition processes and loss of them lead to neuropathologic issues in DS. Even some new investigations restricted the DSCR region to a very small area, duplicated in all DS patients (14). Hence, consideration of lower number of genes, which are more specific for DS phenotypes, seems to be more logical for modifications to correct adverse phenotypes.

### Principles of X chromosome inactivation

In order to progress on this hypothesis, we need to indicate more detailed facts about the cellular regulators in women X chromosome inactivation process. Generally, there is a natural cellular intelligence for inactivating all the defined extra X chromosome in human cells on the different condition, including a normal woman’s cell or any numerical chromosome disorders like XXX women. Inactivation begins in fetal age by the expression of a non-coding RNA (Xist) on the long arm of target X chromosome. Accumulation and spreading of Xist from X inactivation center (XIC) make transcription silencing take place in other parts of X chromosome. The facts indicate the essential role of chromatin modifications and epigenetic changes in gene silencing on Xi (15, 16). Even the best way for the modifications of gene expression and activation could be the design and creation of new epigenetic changes, which can act as an on/off switch for it, not making the alteration in gene sequence itself (17). Therefore the identification and administration of cell products participated in chromatin modifications, epigenetic marks in different cells, and performing the inactivation procedure, would be essential for understanding the inactivation mechanisms and possible use of them as a potential modification tool.

### Short overview on CRISPR/Cas9 system

Using CRISPR/Cas9 system to cut the human genome was developed in early 2013 (18–20). Then lots of publications appeared in this field, introducing various functions for different types of the system. The main utilities could be summarized as 1. Targeting a specific sequence and cutting relevant site, 2. Targeting, cutting the position and replacing it with desired sequence, 3. Attachment of the broken/mutated Cas9 enzyme to the favorite sequence and blocking the transcription or silencing the relevant gene by limiting the RNA polymerase activity, 4. Attachment of the inactive enzyme to an activator protein with a duty on stimulating gene expression results in controlled gene switch on/off (using different RNA guide for different cell lines), 5. Rewriting and making new epigenetic alterations in specific sites by broken/mutated Cas9, 6. Identification of gene function and regulatory regions in genome, and 7. Produce new animal models for various diseases by creating mutations using the CRISPR/Cas9 tool (21–24). CRISPR system aims its target by a single-stranded guide complementary RNA (sgRNA), replaced with synthetic exogenous RNA, and cut the relevant sequence by Cas9 enzyme. The generated double-stranded break (DSB) position can be treated using two alternative ways: a) the system deletes or inserts nucleotides randomly, using Non-Homologous End Joining (NHEJ) repair mechanism, which can lead to target gene sequences disruption. b) insert an external DNA as a template, with homology sequences between both 5’ and 3’ sides of the template and wild type target sequence, for mutation correction in DSB site. The latter is called Homology-Directed Repair (HDR), which can result in the alteration of target gene sequence and function (19, 25). In this way, researchers can perform an extensive range of sequence changing from a single nucleotide to a large insertion or replacement, which would be a permanent alteration in the genome too (26). The gRNA is always designed in a way to introduce DSB to the desired sequence (21). Moreover, just one suitable gRNA would be enough for making a DSB in target sequence and subsequent HDR, without any consideration for insert size (27). Even by using a number of gRNA simultaneously, DSB in large fragments of the genome could be achieved.

### In-vivo delivery of CRISPR/Cas9 for therapeutic purposes

The next important step is introducing a practical way for in vivo delivery purposes. Different types of CRISPR/CAs9 system introduced for transferring into the target cells, comprising Cas9 gene + gRNA gene (gene-based transferring method), Cas9 mRNA + synthetic gRNA (RNA based delivery method), and Cas9 protein + synthetic gRNA. The latter produces a RNP complex and is considered as a protein-based delivery method, which shows many advantages upon two other forms. This type of delivery system demonstrates a lot of benefits i.e. very low off-target ratio, absence of any immunogenicity, no risk for insertional muatgenesis (as it could happen with Cas9 gene transferring to the cell), and more efficiency in editing task (28). However, direct transporting of the Cas9 protein plus other components including gRNA and template DNA into the target cells should utilize a confident and specific approach. Today, nano carriers are proved to be the best way for delivering the CRISPR/Cas9 components in cells. Nanoparticle (NP) vector, coated with cationic polymer (polyethylenimine), has been used for transferring the Cas9 protein and gRNA in cancer model mouse before (29). Moreover, engineered lipid base NPs optimized for specific delivery, applied to convey CRISPR components with high-efficiency percentage (30).

### The Hypothesis and study design

**Fig. 1:**
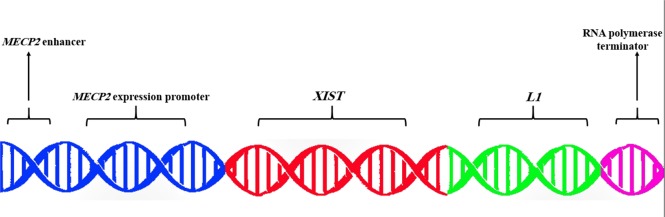
The designed DNA construct for replacing specific, nonfunctional position on Chr21. This includes an enhancer for brain cells special promoter (i.e. *MECP2* promoter), *MECP2* expression promoter, *XIST* gene as a main epigenetic modifier factor for inducing inactivation on relevant chromosome, and a long non-coding RNA gene like *L1* gene on behalf of a particular element for promoting Xist RNA function. Also, an RNA polymerase terminator gene would be necessary in order to control the transcription activities

Here we explore the hypothesis of applying two distinct CRISPR/Cas9 systems simultaneously in a protein-based delivery method to both brain cell culture and DS mouse model. Selecting the brain cells is unavoidable since investigations indicate that most of the genes in DSCR area show a role in learning and cognition processes. Additionally, a mouse model of the disease is the second choice for examination of our theory. The expression and silencing of genes in the inactivated X chromosome of the mouse is controlled by single genes (31). This makes the mouse a suitable model for our inquiry since we consider some specific genes for silencing of other single genes (Table 1). In our opinion, for modification of the DS and attenuation of the main intellectual disability effects, we can inactivate the extra Chr21 in some specific tissues in patients. For this purpose, we assume using two different manipulating CRISPR/Cas9 tools including 1. A Cas9 protein + two different sgRNAs for cutting off two ends of DSCR region in Chr21 (gene deletion approach) and 2. A Cas9 protein + a sgRNA + a template dsDNA to cut a nonfunctional region of the centromere-proximal half of 21q (32), and replace it with a newly designed DNA construct, which contains some major genes in chromatin remodeling and epigenetic effects for the inactivation of one extra Chr21 (Fig. 1).

**Table 1:** List of expressed and silenced Genes on extra chromosome 21 in Down Syndrome patients

**Genes/Sequences**	**Should be expressed**	**Should be silenced**
*XIST*	Aggregates histonic changes, which prevent transcription events through the chromosome (main epigenetic effect)	-
*L1*	Overexprresion of this gene is involved in Xist RNA distribution through the chromosome	
*MECP2* expression promoter	Selective expression of favorite genes in neurons	
*MECP2* enhancer	Enhancer for transcription from *MECP2* promoter	
RNApol terminator	Termination of transcription of favorite genes	
*DSCR1*	--	Involved in DS critical region
*RCAN1*		Involved in DS critical region
*PCP4*	-	Involved in DS critical region
*TTC3*	-	Involved in DS critical region
*MNB*		Involved in DS critical region
*SOD*	-	Involved in DS critical region
*ETS2*	-	Involved in DS critical region
*SIM2*		Involved in DS critical region
*DYRK1*	-	Involved in DS critical region
*DSCAM*		Involved in DS critical region
Other DSCR ^2^ genes^3^		Involved in DS critical region
Genes within *BCE1* and *MX1*^4^		Involved in DS critical region

1. DS: Down syndrome,

2. DSCR: Down Syndrome Critical Region,

3. Refer to Xiang-dong Kong et al. 2014 (51) and Das D. 2014 (5),

4. The gene within this region is shown to be involved in Down syndrome

The latter represents a gene insertion or gene knock-in system by HDR method for inactivating procedure of Chr21. In this way, first, the DSCR region would be omitted and then by insertion of the regulatory DNA construct, whole Chr21 would be exposed to inactivation. In fact, repressing of extra Chr21 and its components would be guaranteed by two subsequent hits. “Make assurance double sure”! We expect inactivation process to occur in other chromosomes too because females′ X chromosome is not the only inactivated chromosome example in human body. Investigations demonstrated that the inactivation also could be seen in males’ germ cells as meiotic sex chromosome inactivation (MSCI) form (33).

Table 2 indicates some key observations in previous studies, which support the feasibility of the hypothesis. Moreover, we proposed the applying of a surface engineered Poly (D, L-lactide-co-glycolide) (PLGA) for delivery of the treatment package (Cas9 + sgRNAs and template DNA) in the brain cells. We discuss the PLGA and other NP carrier’s properties in detail in next section.

**Table 2:** List of studies, which provide evidences for supporting the hypothesis

***Reference***	***Research results***
Song R. et al. 2009	Inactivation can be seen in other chromosomes too
Li et al. 2015	Knock-in of full exon 44 in *DMD* gene by CRISPR methodswith 75% efficiency
Ousterout et al. 2015	All the region between exons 45 to 55 in *DMD* gene have been cut with CRISPR successfully *(this was a relatively large amount of DNA, omitted from the genome)*
Yin H. et al. 2014	Efficiency of 1 in 250 cells is detected by using non-viral delivery methods for CRISPR system
Mout R. et al. 2017	High efficiency for delivery of CRISPR/Cas9 components with Nano-carriers reported
Slaymaker I.M. et al. 2016 and Kleinstiver B.P. et al. 2016	Engineered Cas9 with high specificity for target cells and reduced off-target effects described
Yang Su et al. 2017	CRISPR/Cas9 editing alleviates Huntingon’s symptoms in model mice

## Results and Discussion

The designed DNA construct in this hypothesis, induce the inactivation procedures for extra Chr21 in the patients. The inactivation will follow and portray the events, which occur for extra X chromosomes in females. This, activate a process, which rewrites the epigenetic marks for specific tissue. Here we utilize a *XIST* gene in our construct because it will stimulate the aggregation of histonic changes for expanding prevention of transcription events in whole relevant chromosome (34, 35). Moreover, using a particular long non-coding RNA (lncRNA) like Line-1 (L1) would be beneficial and strengthens Xist function, as previous studies depict the expression and even overexpression of some lncRNAs which modifies the distribution of Xist RNA in relevant chromosome (16, 36). Chromatin analysis methods are available for detection of genes, escaped from inactivation system and could be done after our induced inactivation accomplished (15). Moreover, new approaches like chromosome specific cDNA-array have been used for this purpose in mouse brain cells in recent years (37, 38). Here, we need some comprehensive and accurate techniques for evaluation of inactivation in targeted additional Chr21 in the experiment. Nowadays, different methods including differential expression analysis according to cDNA microarray expression data, Spearman correlation coefficient for analysis of modification in genes and pathways (https://sysbiowiki.soe.ucsc.edu/) (39), and wide gene expression analysis for genes on Chr21 in DS patients (40, 41).

Next, we should employ a brain cell culture media and a DS mouse model for examination of the designed construct. There are some standard brain cells media that are routinely used in experiments (42, 43). However, more advanced results could be achieved if brain cells, obtained from brain biopsy of DS patients, are directly used in cell culture media (21). For finding the suitable and appropriate mouse model of DS, we can refer to J. Braudeau et al. and Miyamoto K. publications (44, 45). After that, sending the modification materials to the brain cells must be accomplished. Generally, intraperitoneal injection is an effective approach for brain cells targeting in mouse model (46).

Subsequent effort will be allocated for entering the appropriate concentrations of Cas9 protein and its properties to the target cells. Recently, engineered Cas9 containing an oligo glutamic acid tag (E-tag), with negative charge has been packaged with sgRNA and are entered in a Gold nanoparticle (AgNP) for direct transfer into the cytoplasm and nucleus space of host cells. The in vitro efficacy was reported for 90% of cells (47). Moreover, engineered Cas9 with high specificity for target cells and reducing off-target effects has been used before (48, 49). Protein-based CRISPR delivery displays low off-target rate in experiments. Finally, by the administration of an appropriate dosage of doxycycline component, Cas9 activation can be controlled in cells (50).

These and many other efforts in this field, show the extreme progress in CRISPR and Nano carrier-mediated methods for human body cells manipulation (Table 2). Here, we recommend using two distinct CRISPR components for complete inactivation achievement. Theoretically, it would be easier and less difficult in laboratory to just aim, cut, and omit the overexpressed genes in DS cells instead of employing an extra Cas9 system with template DNA, which utilizes HDR process for replacing new genes. However, investigations have shown creating some DSB in DNAs without replacing any material, increasing the chance of unwanted chromosomal rearrangements (21). Hence, advanced manipulation methods just like this hypothesis and using new discoveries in cell reprogramming control like adding *ZSCAN4* gene in the construct, would be essential to guarantee the efficacy rate of the system.

Moreover, in this hypothesis, we must get the interfering tools to specific cells in the brain. Using special NPs like Solid lipid Nano particles (SLNs), PLGA, and etc. are the suitable Nano carriers for brain cells delivery (51, 52). Modified and engineered surfaces of these and other NPs can perform the targeting process more specifically. For example, altered surface of Poly (n-butyl cyanoacrylate) with polysorbate 80, demonstrated its ability to pass through blood-brain barrier invivo (53). Recent studies also use the PLGA, which when engineered with natural cell-membrane derived lipid vesicles (nanoghosts), are more successful target specificity for the carrier (54). These investigations on delivery rate of Nano assemblies show that engineering NPs with particular peptides or lipids on their surfaces can guarantee the specific delivery to the target tissues, both in vitro and in vivo.

## Conclusion

This hypothesis proposes a knowledge-based approach to attenuate DS main effects in suffered patients. Besides, the introduced technique has the ability to focus on some specific genes like those involved in cardiac and septal defects and even cancer-prone genes in DS patients (55). Future steps can be modifying of the particular alleles for DS genes in different populations and ethnic groups. This approach brings new hope for DS patients, especially those, who indicate no brain dementia or show dementia in their youth or middle age and promises a better life for them. In addition, main future efforts would be focused on the entering the appropriate dosage of the materials, especially the Cas9 protein, and using the comprehensive assessment methods and functional evaluations of the entire genome after the treatment, by means of wide-ranging techniques like whole genome sequencing (WGS) and other assays.

## Ethical considerations

Ethical issues (Including plagiarism, informed consent, misconduct, data fabrication and/or falsification, double publication and/or submission, redundancy, etc.) have been completely observed by the authors.
